# The Contribution of SPECT/CT in the Diagnosis of Stress Fracture of the Proximal Tibia

**DOI:** 10.4274/mirt.27247

**Published:** 2018-02-01

**Authors:** Berna Okudan, Nazım Coşkun, Pelin Arıcan

**Affiliations:** 1 University of Health Sciences, Ankara Numune Training and Research Hospital, Clinic of Nuclear Medicine, Ankara, Turkey

**Keywords:** Stress fracture, proximal tibia, bone scintigraphy, single-photon emission computed tomography/computed tomography

## Abstract

Stress fractures are injuries most commonly seen in the lower limbs and are usually caused by repetitive stress. While the distal and middle third of the tibia is the most frequent site for stress fractures (almost 50%), stress fractures of the proximal tibia is relatively rare and could be confused with other types of tibial fractures, thus altering management plans for the clinician. Early diagnosis of stress fractures is also important to avoid complications. Imaging plays an important role in the diagnosis of stress fractures, especially bone scan. Combined with single-photon emission computed tomography/computed tomography (SPECT/CT) it is an important imaging technique for stress fractures in both upper and lower extremities, and is widely preferred over other imaging techniques. In this case, we present the case of a 39-year-old male patient diagnosed with stress fracture of the proximal tibia and demonstrate the contribution of CT scan fused with SPECT imaging in the early diagnosis of stress fracture prior to other imaging modalities.

## Abstract (tr)

Stres kırıkları çoğunlukla alt ekstremitede görülen ve genellikle tekrarlayan strese bağlı gelişen yaralanmalardır. Tibia orta kesimi ve distali stres kırıklarının en sık görüldüğü bölgeler iken (yaklaşık %50), proksimal tibianın stres kırıkları nispeten nadirdir, diğer kırık türleriyle karıştırılabilir ve bu durum klinisyenin tedavi planını etkileyebilir. Stres kırıklarının erken tanısı komplikasyonları önlemek açısından önemlidir. Görüntüleme yöntemleri, stres kırıklarının teşhisinde önemli bir rol oynamaktadır. Özellikle tek-foton emisyon bilgisayarlı tomografi/bilgisayarlı tomografi (SPECT/BT) ile birleştirilen kemik sintigrafisi hem üst hem de alt ekstremite stres kırıkları için önemli bir görüntüleme tekniğidir ve diğer görüntüleme tekniklerine göre çok daha fazla tercih edilmektedir. Bu yazıda proksimal tibiada stres kırığı tanısı alan 39 yaşındaki bir erkek hastanın hikayesini sunuyor ve stres kırığının erken tanısında SPECT/BT taramasının katkısını gösteriyoruz.

## Figures and Tables

**Figure 1:**
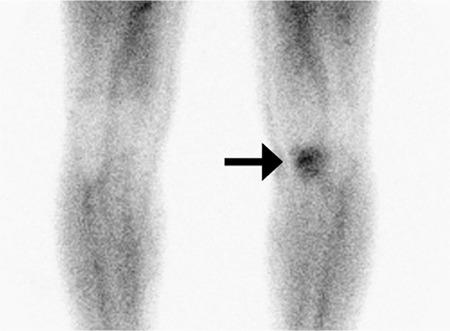
Blood pool phase of 3-phase bone scintigraphy (3-PBS) showing hyperemia in the left knee joint. 3-PBS, revealing the pathologic changes in osseous compartments as early as a few days after the onset of complaints, is a widely used method in the diagnosis of stress fractures.

**Figure 2:**
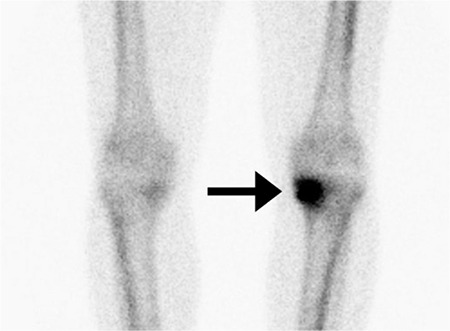
Delayed phase 3-PBS showing increased activity in the left knee joint.

**Figure 3:**
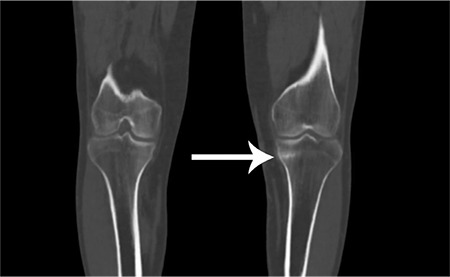
Computed tomography (CT) image from single-photon emission CT (SPECT)/CT fusion study showing a linear fracture in the proximal tibia. While distal and middle third of the tibia are the most frequent sites for stress fractures (almost 50%), stress fractures of the proximal tibia is relatively rare and could be confused with other types of tibial fractures (1). SPECT/CT, which combines the high anatomical resolution of CT with the early-diagnosis capability of SPECT, is an increasingly preferred imaging technique in the diagnosis and follow-up of these patients (2).

**Figure 4:**
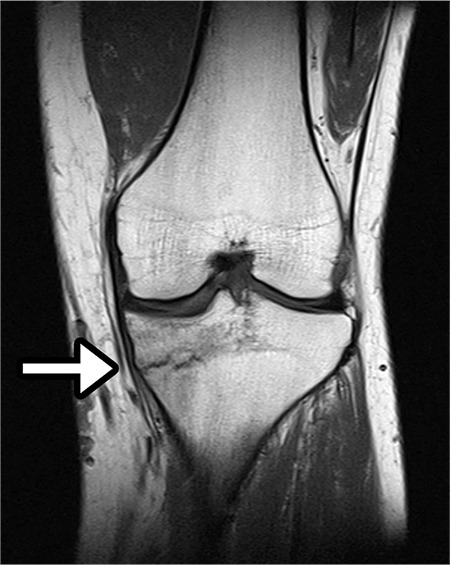
Magnetic resonance image confirming the stress fracture line in the proximal tibia.
